# Molecular characterization of nosocomial *Clostridium difficile* infection in pediatric ward in Iran

**DOI:** 10.1186/s40064-015-1268-0

**Published:** 2015-10-19

**Authors:** Abolfazl Khoshdel, Roya Habibian, Neda Parvin, Abbas Doosti, Fatemeh Famouri, Ali Eshraghi, Massoud Hafizi

**Affiliations:** Biochemistry Research Center, Shahrekord University of Medical Sciences, Shahrekord, Iran; Department of Infectious Diseases, Shahrekord University of Medical Sciences, Hajar Hospital, Parastar Street, Shahrekord, Iran; Nursing Department, Shahrekord University of Medical Sciences, Shahrekord, Iran; Biotechnology Research Center, Islamic Azad University, Shahrekord Branch, Shahrekord, Iran; Department of Pediatrics, Isfahan University of Medical Sciences, Isfahan, Iran; Department of Pediatrics, Shahrekord University of Medical Sciences, Shahrekord, Iran; Department of Infectious Diseases, Shahrekord University of Medical Sciences, Shahrekord, Iran

**Keywords:** *Clostridium difficile*, Diarrhea, Pediatrics, Ribotyping, Iran

## Abstract

*Clostridium difficile* is recognized as a major cause of nosocomial acquired antibiotic-associated diarrhea and pseudomembranous colitis. It is a significant financial burden on modern healthcare resources. This study aimed to assess the molecular characterization of *C. difficile* strains isolated from children under 5 years old suffered from nosocomial diarrhea. One hundred diarrheic and 130 non-diarrheic fecal samples were collected from pediatrics less than 5 years old. Samples were cultured and *C. difficile* isolates were subjected to the PCR technique to study the distribution of ribotypes of *C. difficile* using P3 and P5 primers. Fifty-two out of 100 samples (52 %) were positive for *C. difficile*. The prevalence of bacterium in healthy children was 4.61 %. Total prevalence of *C. difficile* in diarrheic girls and boys were 48.9 and 54.7 %, respectively. Thirteen to twenty-four month age children had the highest prevalence of *C. difficile*. The most commonly detected ribotypes in the *C. difficile* isolates of Iranian pediatrics were RT027 (11.52 %), R1 (9.61 %) and R13 (7.68 %). The ribotypes of all of the six bacterial isolates of healthy children was not diagnosed. According to the presence of *C. difficile* and R27 ribotype, a continued genotype surveillance of this bacterium is necessary to monitor changes in the prevalence of certain strains and to identify the emergence of new strains that could affect future vaccine strategies.

## Background

Diarrhea is one of the most common diseases in children in developed and developing countries. It has been documented that there are about two billion cases of diarrheal disease worldwide every year and 1.9 million children younger than 5 years of age hospitalized due to diarrhea each year (Farthing et al. 2013). It has also been recorded that more than 5000 children are dying every day as a result of diarrheal diseases (Farthing et al. 2013). One of the most prevalent cause of diarrhea in children less than 5 years old is *Clostridium difficile* (*C. difficile*) (Wilson 2006; Koletzko and Osterrieder 2009). *C. difficile* is a spore-forming, anaerobic, gram-positive bacillus an etiologic agent of nosocomial diarrhea and pseudomembranous colitis. Disease is mainly occurred in children who have been treated with long term antibiotics (Brown et al. 2015; Min Cho et al. 2012).

Changes in the epidemiology of *C. difficile* infections in the pediatric population is a serious concern. However, benign neonatal colonization with toxigenic *C. difficile* is a well-documented phenomenon but recent studies have suggested an increased incidence of *C. difficile* associated diarrhea (CDAD) in children (Cohen et al. 2006; Gleizes et al. 2006). Previous investigation which was conducted on 22 hospitals in the United States reported an increased prevalence of CDAD in children (increased by 53 % from 2001 to 2006, with 26 % of patients with CDAD ≤1 year of age) (Cohen et al. 2006).

Polymerase chain reaction (PCR)-based ribotyping of *C. difficile* in clinical samples is a good approach to identify the novel aspects in the epidemiology of diarrhea caused by this bacterium in children (Arvand et al. 2009; Krutova et al. 2014). High prevalence of *C. difficile* ribotype 027 (RT027) in the clinical samples of pediatric patients have been reported in various studies (Arvand et al. 2009; Krutova et al. 2014; Oleastro et al. 2014). Diarrheal disease caused by this ribotype of the *C. difficile* is more severe, with higher rates of morbidity and mortality and more virulent (Arvand et al. 2009; Krutova et al. 2014; Oleastro et al. 2014).

According to the high potential of *C. difficile* in causing infection, diarrhea and various complications in pediatrics, typing of this bacterium is essential to understand the exact epidemiology of outbreaks, identify any possible incidence of cross infection and to set up surveillance programs to monitor virulent strain emergence and hospital reservoirs. However, few studies have been addressed in this field in Iran. Therefore, the present study was carried out in order to study the prevalence of *C. difficile* and its ribotypes in Iranian pediatric patients suffered from nosocomial diarrhea.

## Results and discussion

Between zero to three antibiotics were used by patients with average 1.42 ± 0.68. Four patients did not take any antibiotic. Fifty-seven patients received one, thirty-two received two and seven received three types of antibiotics. There were no antibiotic usage in the control group. Gentamycin, ceftriaxone, ampicillin, erythromycin, clindamycin and vancomycin were used in the pediatric patients of this study. The treatment periods by antibiotics were different according to the type of infection. The average age of patients was 11.8 ± 15.3 months (range 6–60 months), and 47 % were female. The average weight of patients was 9 ± 2.7 kg. Frequency of *C. defficile* based on each sex and age groups is shown in Table 1. Chi Square test didn’t show any significant difference for the distribution of *C. defficile* based on sex and age group of children. The most commonly used antibiotic agent was ceftriaxone (93 %). There were no significant relation between positive results with antibiotic treatment.

**Table 1:** Prevalence of *C. defficile* based on sex and age groups

Groups of children	Variable	Group	Positive		Negative		Total		P value
			Number	Percent	Number	Percent	Number	Percent	
Patients	Sex	Girl	23	48.9	24	51.1	47	100	0.564
		Boy	29	54.7	24	45.3	53	100	
	Age	6–12 months	30	50	30	50	60	100	0.814
		13–24 months	16	57.1	12	42.9	28	100	
		>24 months	6	50	6	50	12	100	
Healthy	Sex	Girl	2	3.3	58	96.6	60	100	0.225
		Boy	4	5.71	66	94.2	70	100	
	Age	6–12 months	–	–	58	100	58	100	
		13–24 months	1	2.5	39	97.5	40	100	0.198
		>24 months	5	15.6	27	84.3	32	100	0.312

Electrophoresis of PCR products revealed 5–6 fragments with 260–600 bp(s) length which showed the distribution of *C. difficile* ribotypes. From 100 examined samples, 52 (52 %) were positive for *C. difficile*. Total prevalence of *C. difficile* in girls and boys patients were 48.9 and 54.7 %, respectively. Thirteen to twenty-four month age children had the highest prevalence of *C. difficile*. Of 130 fecal samples of healthy children, 6 samples (4.61 %), were positive for *C. difficile*. Boys had the higher prevalence of *C. difficile* than girls. Older than 24 months healthy children had the highest prevalence of *C. difficile* (15.6 %). There were statistically significant differences (p < 0.05) for the prevalence of the *C. difficile* between healthy and sick groups. The most commonly detected ribotypes in the *C. difficile* isolates of hospitalized children were RT027 (11.52 %), RT01 (9.61 %) and RT013 (7.68 %) (Table 2). Totally, the ribotypes of the 13.44 % of *C. difficile* isolates of hospitalized children and all of the healthy children were not recognized.

**Table 2:** Distribution of ribotypes of *C. difficile* in the stool specimens of hospitalized and healthy pediatric of Iran

Ribotypes	Total distribution in hospitalized children		Total distribution in healthy children	
	Number	Percent (%)	Number	Percent (%)
R1	5	9.61	–	–
R7	2	3.84	–	–
R8	2	3.84	–	–
R12	3	5.76	–	–
R13	4	7.68	–	–
R15	3	5.76	–	–
R18	3	5.76	–	–
R27	6	11.52	–	–
R28	2	3.84	–	–
R29	3	5.76	–	–
R31	3	5.76	–	–
R32	1	1.92	–	–
R81	1	1.92	–	–
R82	1	1.92	–	–
R83	2	3.84	–	–
R86	1	1.92	–	–
R87	2	3.84	–	–
R88	1	1.92	–	–
^a^	7	13.44	6	100
Total	52	100	6	100

^a^Unknown ribotype

*C. difficile* was the most common agent observed in 52 % of episodes associated with nosocomial diarrhea in this pediatric population. The prevalence of *C. difficile* nosocomial diarrhea was varied in different studies, but there is limit studies in this field in Iran. Sadeghifard et al. (2010) were analyzed a total of 942 stool samples from Iranian patients with nosocomial diarrhea. They showed that 57 samples (6.1 %) were positive for toxigenic *C. difficile* (Sadeghifard et al. 2010). One possible difference between our results and the results of Sadeghifard et al. (2010) was the fact that they found significant differences (p < 0.05) between the rate of isolated toxigenic *C. difficile* and age of patients (Sadeghifard et al. 2010). Miller et al. (2002) assessed the healthcare burden, morbidity, and mortality of nosocomial *C. difficile*-associated diarrhea (N-CDAD) in Canadian hospitals. They found that 371 out of 2062 tested samples (18 %) were positive for *C. difficile* and 269 strains of total 371 positive isolates (13 %) met the case definition for nosocomial CDAD (Miller et al. 2002). In the survey which was conducted by Langley et al. (2002), *C. difficile* (39 of 122; 32 %) was one of the most common pathogens in nosocomial diarrhea episodes (Langley et al. 2002) which was similar to our findings. In a study carried out by Gursoy et al. (2007), the total prevalence of *C. difficile* was 27.7 % and the most commonly used antibiotic was clindamycin (Gursoy et al. 2007).

Direct detection of *C. difficile* toxin genes in stool samples by real-time PCR has a sensitivity superior than stool culture EIAs and performance comparable to that of real-time PCR assay of cultured isolates. Luna et al. (2011) found that the sensitivities of stool real-time PCR and stool EIA were 95 and 35 %, respectively, with a specificity of 100 % for both methods (Luna et al. 2011).

In our study, majority of patients with *C. difficile* infections received antibiotics. One of the most frequent complications of antibiotic therapy is diarrheal syndrome, which may be related both to the direct toxic effects of antibiotics on the gastrointestinal tract, and the activation of conditionally pathogenic intestinal flora. Therefore, particular attention should perform on the problem of *C. difficile* infections, which may be the consequence of the development of pseudomembranous colitis in both children and adults (Zakharova et al. 2011).

Antimicrobial agents are the most frequently prescribed medicines in children because of acute infectious diarrhea. The previous study which was conducted on the pediatric patients of same hospital represented that the total prescription of antibiotics in an irregular and inappropriate manner was 37 % (Khoshdel and Panahandeh 2012). Therefore, it is not surprising that the prevalence of *C. difficile* in the pediatric patients of our study was 52 %. Types of antibiotics used for treatment of cases of diarrhea can influence on the prevalence of *C. difficile*. It is because of the medical practitioners do not use inexpensive and easy assays like the disk diffusion method for antibiotic prescription. In fact, the experience of medical practitioners in the treatment of previous cases of diseases, is their main reason for antibiotic prescription. It is clear that the previous experiences of practitioners are not sufficient for treatment of the novel cases of diseases like diarrhea. Therefore, prescription of antibiotics in a highly irregular manner and without accordance to the results of disk diffusion method, caused occurrence of antibiotic resistance in bacteria like *C. difficile*. Therefore, presence of the resistant strains of this bacterium will increase. Low prevalence of this bacterium in healthy children which didn’t receive any antibiotics has indirectly confirmed the relationship between antibiotic prescription and occurrence of *C. difficile* infections.

Another part of our investigation has focused on the determination of ribotypes of *C. difficle*. Result showed that the most commonly detected ribotype in the *C. difficile* isolates of our investigation was RT027. Prior to 2003 year, only a handful of these strains were isolated in the United Kingdom, whereas currently most typed isolates are RT027. This is also mirrored in Canada, where RT027 strains were undetected in 2000, but reached 75.2 % of all PCR-ribotype strains in 2003 (MacCannell et al. 2006). Kuijper et al. (2008) reported that the RT027 of the *C. difficile* was the most commonly detected ribotypes in the cases of severe complicated diarrhea. High prevalence of the RT027 strains of *C. difficile* have been reported in majority of European countries such as Germany, France, Belgium, Ireland, Finland, Poland, Spain, Hungary, the Netherlands, Switzerland, Luxembourg and also the United Kingdom (Kuijper et al. 2008). Oleastro et al. (2014) reported that 72.72 % of the *C. difficile* isolates of pediatrics in Portugal were positive for the RT027 which was entirely high.

## Methods

### Ethical considerations

This research project has been approved by the ethical committee of Shahrekord University of Medical Sciences, Iran. Informed consent was obtained from pediatrics or their parents.

### Samples collection and bacterial isolation

This cross-sectional study was conducted from December 2013 to May 2014. The studied population comprised all patients, aged 6–60 months, who had been hospitalized in the pediatric ward of Hospitals because of diseases other than diarrhea such as urinary tract infection, bacterial pneumonia, acute respiratory illness and skin infections. Fecal specimens were collected from 100 children with clinical signs of nosocomial diarrhea admitted in the hospitals and health centers of south west of Iran. Nosocomial diarrhea was defined as diarrhea occurring more than 72 h after admission to hospital for non-diarrheal causes. The fecal samples were transported daily to the Cellular and Molecular Research Center, Shahrekord University of Medical Sciences in refrigerated boxes using the Cary-Blair transport (Para-Pak™ C&S transport media; Meridian Diagnostics, Cincinnati, OH, USA) transitional media. The fecal samples of 130 healthy children with similar criteria (male and female, aged 6–60 months) were also collected for the control group.

Five gram of each sample was transferred to 20 mL of *C. difficile* broth (CDB; Oxoid SR0048) containing 40 g/l proteose peptone, 5.0 g/l disodium hydrogen phosphate, 0.1 g/l magnesium sulphate, 2.0 g/l sodium chloride, 6.0 g/l fructose and 1.0 g/l sodium taurocholate supplement with *C. difficile* selective supplement (Oxoid, UK, Code: SR0173) and 5 % (v/v) defibrinated sheep blood. After incubation at 37 °C for 2 days under anaerobic conditions, 2 mL of the enrichment broth was added to 2 mL of 96 % ethanol in a centrifuge tube and homogenized for 50 min on a shaker at room temperature. After centrifugation (3800×*g* for 10 min), a loopful of the sediment was streaked onto cycloserine cefoxitin fructose agar (CCFA) containing sodium taurocholate. All plates were incubated in an anaerobic chamber (Don Whitley Scientific Ltd., Shipley West Yorkshire, UK) at 37 °C for 48 h. Colonies of *C. difficile* were identified on the basis of their characteristic colony morphology (yellow, ground glass appearance), Gram staining, odour (horse dung smell), their chartreuse fluorescence under long-wave UV light (~360 nm) and l-proline aminopeptidase test (Harvey et al. 2011).

### Ribotyping of *C. difficile*

Genomic DNA was extracted from typical colonies of *C. difficile* using genomic DNA extraction Kit (Fermentase, Germany) according to the manufacturer’s recommendation. The isolated DNA was quantified by spectrophotometric measurement at a wave length of 260 nm according to the method described by Sambrook and Russell (2001). The extracted DNA of each specimen was kept frozen at −20 °C until used. Colonies of *C. difficile* were approved another one using the real-time PCR method described by Bélanger et al. (2003).

### PCR ribotyping

Ribotyping was done in the Biotechnology Research Center of the Islamic Azad University of Shahrekord Branch, Iran. PCR technique was used to study the distribution of ribotypes of *C. difficile*. The oligonucleotide primers p3-F: 5′-CTGGGGTGAAGTCGTAACAAGG-3′ (position 1445–1466 of the 16S rRNA gene) and p5-R: 5′-GCGCCCTTTGTAGCTTGACC-3′ (position 20–1 of the 23S rRNA gene) described by Stubbs et al. (1999) were used for gene amplification. *C. difficile* 027/NAP1/BI strain was used as a positive control. A negative-DNA control was performed by adding 1 μL of sterile ultrapure deionized water. For investigation of ribotypes of *C. difficile* the specimens were amplified in a Gradient Palm Cycler (Corbett Research, Australia) and PCR was performed in a total volume of 25 μL including 0.5 ml tubes containing 1 μg of genomic DNA, 1 μM of each primer, 2 mM MgCl_2_, 200 Μm dNTP, 2.5 μL of 10× PCR buffer and 1 unit of Taq DNA polymerase (Fermentas, Germany). PCR cycles consisted of an initial denaturation step (95 °C for 5 min) followed by 30 amplification cycles (denaturation at 94 °C for 1 min, annealing at 62 °C for 1 min, and elongation at 72 °C for 1 min) with a final elongation at 72 °C for 5 min and amplified samples were held at 4 °C.

### Analysis of PCR products

The amplified products were detected in 1 % agarose gel electrophoresis. The electrode buffer was TBE [Tris-base 10.8 g 89 mM, Boric acid 5.5 g 2 mM, EDTA (pH 8.0) 4 ml of 0.5 M EDTA (pH 8.0), combine all components in sufficient H_2_O and stir to dissolve]. Aliquots of 10 µL of PCR products were applied to the gel. Constant voltage of 80 V for 30 min was used for products separation. The DNA fragment size was compared with a standard molecular weight (100 bp DNA ladder of Fermentas, Germany). After electrophoresis, the amplicons were visualized with ultraviolet light after ethidium bromide (5 μg mL^−1^) staining and photographs were obtained in UVIdoc gel documentation systems (UK).

### Ribotyping patterns

Comparison of PCR ribotyping patterns was performed visually. All isolates were typed using the PCR ribotyping method described by Bidet et al. (1999) and Gel Compare software. Strains with ribotype patterns that differed by at least one band were assigned to different types. Ribotype group was designated by upper and lower-case letters combined with a number.

### Statistical analysis

Analysis of data and investigation of ribotypes of *C. difficile* were performed using the SPSS (version 11.5) and Gel Compar software.

## Conclusion

The present study showed a low frequency of nosocomial infection due to *C. difficile* and especially it’s RT027. Our work emphasizes the importance of nosocomial infections in pediatric wards which is important as its considerable costs of hospitalization and treatment. A continued ribotype surveillance of *C. difficile* is necessary to monitor changes in the prevalence of strains, to identify the emergence of new strains over time that could affect future vaccine strategies, and to identify any regional differences of ribotype prevalence. Clinician should pay more attention in prescription of antibiotics in the cases of diarrhea especially in children.

## Abbreviations

*C. difficile*: *Clostridium difficile*
PCR: polymerase chain reaction
CDAD: *Clostridium difficile* associated diarrhea
RT027: ribotype 027

## References

[CR1] Arvand M, Hauri AM, Zaiss NH, Witte W, Bettge-Weller G (2009) *Clostridium difficile* ribotypes 001, 017, and 027 are associated with lethal *C. difficile* infection in Hesse, Germany. Euro Surveill 14(45) 10.2807/ese.14.45.19403-en 19941785

[CR2] Bélanger SD, Boissinot M, Clairoux N, Picard FJ, Bergeron MG (2003). Rapid detection of *Clostridium difficile* in feces by real-time PCR. J Clin Microbiol.

[CR3] Bidet P, Barbut F, Lalande V, Burghoffer B, Petit JC (1999). Development of a new PCR ribotyping method for *Clostridium difficile* based on ribosomal RNA gene sequencing. FEMS Microbiol Lett.

[CR4] Brown K, Valenta K, Fisman D, Simor A, Daneman N (2015). Hospital ward antibiotic prescribing and the risks of *Clostridium difficile* infection. JAMA Intern Med.

[CR5] Cohen SH, Tang YJ, Silva J (2006). Molecular typing methods for the epidemiological identification of *Clostridium difficile* strains. Exp Rev Mol Diagn.

[CR23] Farthing M, Salam MA, Lindberg G, Dite P, Khalif I, Salazar-Lindo E, Ramakrishna BS, Goh KL, Thomson A, Khan AG, Krabshuis J, LeMair A (2013). World Gastroenterology Organization. Acute diarrhea in adults and children: a global perspective. J Clin Gastroenterol.

[CR6] Gleizes O, Desselberger U, Tatochenko V, Rodrigo C, Salman N, Mezner Z, Giaquinto C, Grimprel E (2006). Nosocomial rotavirus infection in European countries: a review of the epidemiology, severity and economic burden of hospital-acquired rotavirus disease. Pediatr Infect Dis J.

[CR7] Gursoy S, Guven K, Arikan T, Yurci A, Torun E, Baskol M, Ozbakir O, Yucesoy M (2007). *Clostridium difficile* infection frequency in patients with nosocomial infections or using antibiotics. Hepato-gastroenterology.

[CR8] Harvey RB, Norman KN, Andrews K, Hume ME, Scanlan CM, Callaway TR, Anderson RC, Nisbet DJ (2011). *Clostridium difficile* in poultry and poultry meat. Foodborne Pathog Dis.

[CR9] Khoshdel A, Panahandeh GHR (2012). The pattern of antimicrobial utilization in patients of pediatric wards in Hajar hospital, Shahrekord, Iran in 2009–2010. J Shahrekord Univ Med Sci.

[CR10] Koletzko S, Osterrieder S (2009). Acute infectious diarrhea in children. Dtsch Arztebl.

[CR11] Krutova M, Matejkova J, Nyc O (2014). *C. difficile* ribotype 027 or 176?. Folia Microbiol (Praha).

[CR12] Kuijper EJ, Barbut F, Brazier JS, Kleinkauf N, Eckmanns T, Lambert ML, Drudy D, Fitzpatrick F, Wiuff C, Brown DJ, Coia JE, Pituch H, Reichert P, Even J, Mossong J, Widmer AF, Olsen KE, Allerberger F, Notermans DW, Delmée M, Coignard B, Wilcox M, Patel B, Frei R, Nagy E, Bouza E, Marin M, Akerlund T, Virolainen-Julkunen A, Lyytikäinen O, Kotila S, Ingebretsen A, Smyth B, Rooney P, Poxton IR, Monnet DL (2008). Update of *Clostridium difficile* infection due to PCR ribotype 027 in Europe, 2008. Euro Surveill.

[CR13] Langley JM, LeBlanc J, Hanakowski M, Goloubeva O (2002). The role of *Clostridium difficile* and viruses as causes of nosocomial diarrhea in children. Infect Control Hosp Epidemiol.

[CR14] Luna RA, Boyanton BL, Mehta S, Courtney EM, Renee Webb C, Revell PA (2011). Rapid stool-based diagnosis of *Clostridium difficile* infection by real-time PCR in a children’s hospital. J Clin Microbiol.

[CR15] MacCannell DR, Louie TJ, Gregson DB, Laverdiere M, Labbe AC, Laing F, Henwick S (2006). Molecular analysis of *Clostridium difficile* PCR ribotype 027 isolates from Eastern and Western Canada. J Clin Microbiol.

[CR16] Miller MA, Hyland M, Ofner-Agostini M, Gourdeau M, Ishak M, Canadian Hospital Epidemiology Committee, Canadian Nosocomial Infection Surveillance Program (2002). Morbidity, mortality, and healthcare burden of nosocomial *Clostridium difficile*-associated diarrhea in Canadian hospitals. Infect Control Hosp Epidemiol.

[CR17] Min Cho S, Joon Lee J, Yoon HJ (2012). Clinical risk factors for *Clostridium difficile*-associated diseases. Braz J Infect Dis.

[CR18] Oleastro M, Coelho M, Gião M, Coutinho S, Mota S, Santos A, Rodrigues J, Faria D (2014). Outbreak of *Clostridium difficile* PCR ribotype 027—the recent experience of a regional hospital. BMC Infect Dis.

[CR19] Sadeghifard N, Salari MH, Ghassemi MR, Eshraghi S, Amin Harati F (2010). The incidence of nosocomial toxigenic *Clostridium difficile* associated diarrhea in Tehran tertiary medical centers. Acta Med Iran.

[CR20] Sambrook J, Russell DW (2001). Molecular cloning: a laboratory manual.

[CR21] Stubbs SLJ, Brazier JS, O’Neill GL, Duerden BI (1999). PCR targeted to the 16S-23S rRNA gene intergenic spacer region of *Clostridium difficile* and construction of a library consisting of 116 different PCR ribotypes. J Clin Microbiol.

[CR22] Wilson ME (2006). *Clostridium difficile* and childhood diarrhea: cause, consequence, or confounder. Clin Infect Dis.

[CR24] Zakharova IN, Dmitrieva IA, Goriukhina OM, Sugian NG (2011). Prevention of antibiotic-associated diarrhea in children. Exp Clin Gastroenterol.

